# Critical Evaluation of Key Methods for RNA Quantification

**DOI:** 10.3390/ijms27167379

**Published:** 2026-08-18

**Authors:** Valentina Paracchini, Mauro Petrillo, Luca Fornara, Sabrina Gioria, Gabriele Leoni, Giuditta Guerrini, Otmar Geiss, Antonio Marchini, Luigi Calzolai

**Affiliations:** 1 European Commission, Joint Research Centre (JRC), 2440 Geel, Belgium; valentina.paracchini@ec.europa.eu; 2 Seidor Italy SRL, 20129 Milan, Italy; mauro.petrillo@seidor.com; 3 European Commission, Joint Research Centre (JRC), 21027 Ispra, Italy; luca.fornara@ec.europa.eu (L.F.); sabrina.gioria@ec.europa.eu (S.G.); gabriele.leoni@ec.europa.eu (G.L.); giuditta.guerrini@ec.europa.eu (G.G.); otmar.geiss@ec.europa.eu (O.G.); antonio.marchini@ec.europa.eu (A.M.)

**Keywords:** mRNA quantification, digital PCR (dPCR), HPLC, UV spectroscopy, LNP-mRNA, analytical techniques, mRNA quality control, mRNA integrity, mRNA drug substance

## Abstract

The approval of safe and effective lipid nanoparticles-encapsulated mRNA (LNP-mRNA) vaccines during the SARS-CoV-2 pandemic is catalysing the development of the next generation of mRNA therapeutics. Accurate characterisation methods are crucial for assessing the quality and efficacy of these complex formulations. Several analytical techniques exist for the quantification of the mRNA drug substance, including UV spectroscopy, capillary electrophoresis, high-performance liquid chromatography (HPLC), and digital PCR (dPCR). Here, we compare these methods, highlight their limitations, and discuss critical considerations for experimental design to improve the agreement between different analytical methods.

## 1. Introduction

Lipid nanoparticles-encapsulated mRNA (LNP-mRNA) formulations have played a pivotal role in the global response to the COVID-19 pandemic, with proven safety and efficacy, and are now at various stages of development for other infectious diseases, cell and gene therapies [1,2,3,4], and anticancer vaccines [5,6,7,8,9]. LNP-mRNA formulations used in SARS-CoV-2 vaccines consist of a lipid nanoparticle (LNP) and the cargo, a ribonucleic molecule, encoding a polypeptide to elicit an immune response, in the case of vaccines, or to treat aberrantly expressed gene(s), in the case of genetic disorders. The quantification of the mRNA molecule (the drug substance, in regulatory terms) is a quality attribute (QA) that needs to be accurately measured [10].

The mRNA molecule usually consists of a 5′ chemical capping, the 5′ untranslated region, the codifying region encoding the polypeptide, a 3′ untranslated region, and a final 3′ poly (A) tail of around 100 nucleotides. In addition, chemical modifications can be introduced into the mRNA molecule (such as N1-Methylpseudouridine, m1Ψ, or 5-Methoxyuridine, 5moU) to reduce immunogenicity, increase stability, and improve efficacy. The quality of the mRNA drug substances is determined by assessing their identity, purity, quantity and integrity. These attributes are critical for ensuring the safety, quality, efficacy, and batch to batch consistency of mRNA-based therapeutics, including vaccines and gene therapies. Key regulatory guidelines, such as those from the European Medicines Agency (EMA) and the European Pharmacopeia, outline stringent requirements for mRNA characterisation [11,12].

To ensure quality and batch-to-batch consistency, accurate quantification of mRNA is essential. For this, different orthogonal methods are available: UV-based methods are the simplest analytical methods to quantify nucleic acids; the absorbance at 260 nm is linked to the amount of material by using the Lambert–Beer law. Capillary electrophoresis (CE) is an effective technique for separating mRNA based on size and charge, allowing for the assessment of integrity by detecting full-length and fragmented species [13]. With proper calibration it can quantify the amount of the different RNA fragments present in the sample. Digital PCR (dPCR) is a powerful and very sensitive technique for absolute quantification of mRNA copies without the need for calibration standards [14]. High-performance liquid chromatography with UV detection (HPLC-UV) can also detect mRNA molecules of different sizes and quantify them with UV absorption [15,16]. Each of these methods has strengths and weaknesses; some of them also offer information about additional quality attributes such as identity and integrity.

In this study, we evaluated widely used techniques for mRNA quantification—UV spectroscopy, capillary electrophoresis, digital PCR (dPCR), and HPLC-UV—by assessing their analytical performance using representative mRNA samples. We employed Firefly Luciferase (FLuc)-encoding mRNA, a well-established model system in nucleic acid analytics and lipid nanoparticle (LNP) formulation studies [17]. We examined two variants of the CleanCap^®^ FLuc mRNA: (i) the unmodified mRNA, containing native uridine bases (mRNA-FLUC); (ii) the 5-methoxyuridine-modified mRNA (5moU mRNA-FLUC), a nucleoside modification (5moU) known to protect mRNA from immune recognition and degradation.

By providing a comprehensive comparison of key quantification techniques, this study aims to support researchers and regulators in establishing robust quality control standards for mRNA-based medicines.

## 2. Results

To evaluate the analytical performance of the four mRNA quantification techniques, we used two different types of Firefly Luciferase (FLuc)-encoding mRNA: (i) the unmodified mRNA (containing native uridine bases); (ii) the 5-methoxyuridine-modified mRNA (5moU-mRNA), a nucleoside modification (5moU) known to increase RNA stability and attenuate undesired immune responses (the same modification found in the BioNTech/Pfizer and Moderna’s COVID-19 vaccine).

Most of the techniques used in this study report the concentration as mass per volume, whereas PCR data are expressed as copy number per unit volume (cp/µL). Both mRNA samples were initially supplied by the manufacturer at a declared concentration of 1 mg/mL. Using the predicted molecular weight of 621 KDa for mRNA-FLUC and 629 KDa for 5moU-FLUC, these concentrations correspond to 9.7 × 10^11^ cp/µL and 9.6 × 10^11^ cp/µL for the two samples.

### 2.1. UV Spectrophotometry

We used two UV-based systems: a traditional cell-based UV-Vis spectrophotometer and a drop-based NanoDrop system. Both rely on measuring absorbance at 260 nm (A_260_) and applying the Lambert–Beer law (A = ε × b × C), where ε represents the molar extinction coefficient and b is the optical path length. Thus, the value of the molar extinction coefficient (ε_260_) is critical for the accuracy of the results. While the standard conversion factor for single-strand RNA (1 OD_260_ = 40 µg/mL for ssRNA) remains widely adopted (for example, it is the default value in the NanoDrop instruments), recent studies have challenged this value. Cavaluzzi and Borer [18] have reported a revised ε_260_ of 37 µg/mL for ssRNA, highlighting potential discrepancies in concentration measurements.

The concentrations listed in Table 1 were calculated using two conversion factors for absorbance at 260 nm: the conventional 1 OD_260_ = 40 µg/mL and the revised 1 OD_260_ = 37 µg/mL. NanoDrop spectrophotometry, which employs the 40 µg/mL factor, gave concentrations of 959 ± 3 µg/mL for the unmodified mRNA-FLUC and 1057 ± 12 µg/mL for the 5moU mRNA-FLUC. Cuvette-based measurements, also using the 40 µg/mL conversion, yielded 1012 ± 5 µg/mL for the mRNA-FLUC and 1268 ± 4 µg/mL for the 5moU mRNA-FLUC. When the revised conversion factor of 37 µg/mL was applied, the concentrations were 936 ± 5 µg/mL for the unmodified sample and 1170 ± 4 µg/mL for the 5moU sample.

These results show that both UV-based techniques yield precise measurements (with less than 5% error). The accuracy is difficult to assess due to the lack of mRNA reference materials, but it strongly depends on the conversion factor applied. Using one of the two suggested conversion factors (40 µg/mL vs. 37 µg/mL) introduces a 7.5% bias in ssRNA quantification, a deviation exceeding the intra-measurement variance.

Another critical consideration is the sample volume required for each method. The NanoDrop system requires only 1 µL of sample in the 1 mg/mL range [19]. In contrast, the cuvette-based spectrophotometer requires at least 400 µL in the tens of µg/mL range. Considering the measurement requirements (sample preparation, operational simplicity, and material volume), the NanoDrop instrument offers a simple, fast and precise solution for routine RNA concentration assessments if the starting mRNA concentration is high enough. On the other side, the cuvette-based UV spectrophotometer may be better suited for regulated environment requiring higher reproducibility andin the case of mRNA samplesat low concentration. While both UV-based techniques provide accurate quantification of the total RNA concentration, they offer no information regarding the identity or integrity of the mRNA samples.

### 2.2. Bioanalyzer (Capillary Electrophoresis)

**Figure 1:**
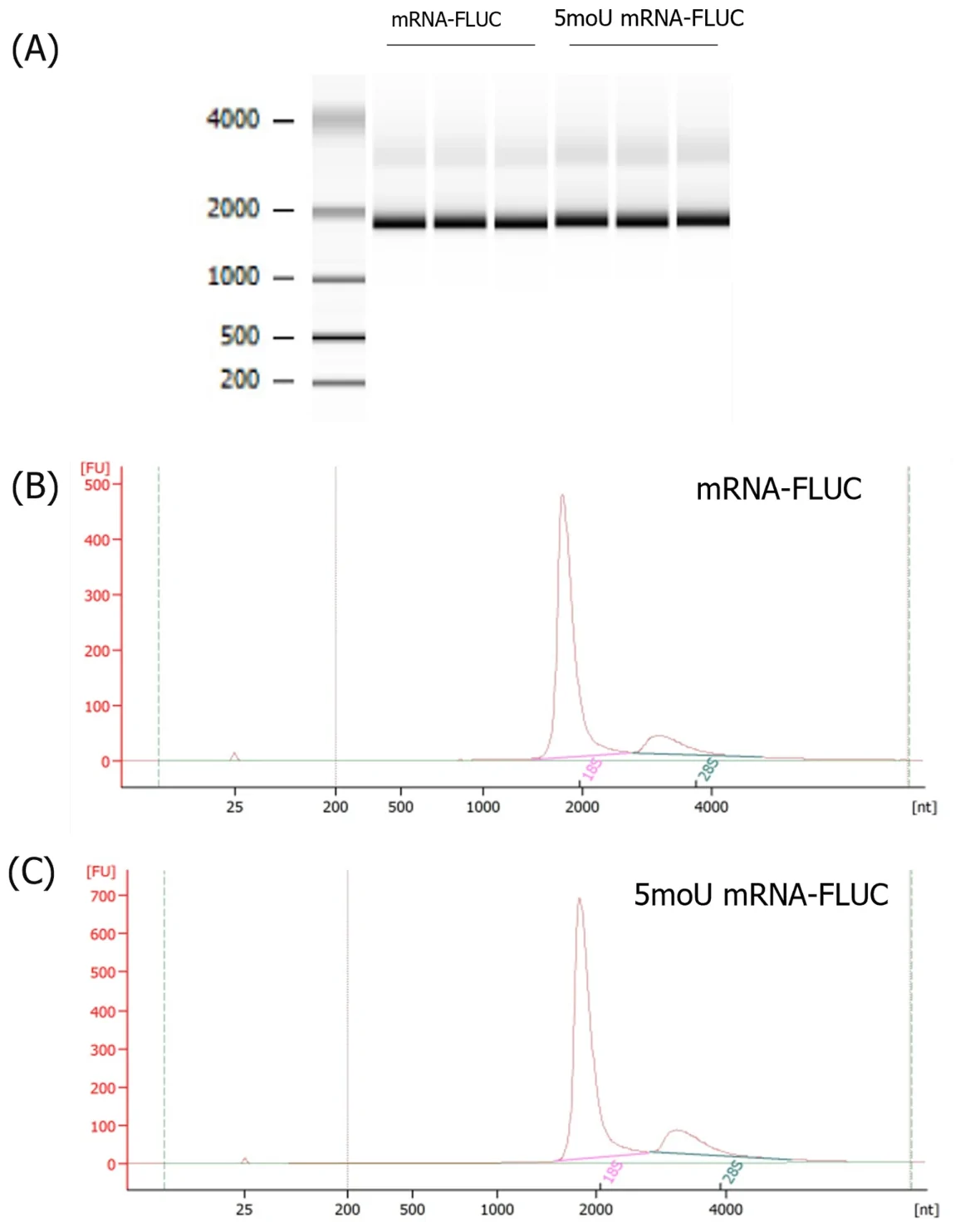
Agilent 2100 Bioanalyzer. (**A**) Gel electrophoresis of the mRNA-FLUC and 5moU mRNA-FLUC run in triplicate; electropherograms of the (**B**) mRNA-FLUC and (**C**) 5moU mRNA-FLUC run.

The two mRNA samples were run on an automated electrophoresis platform. Figure 1A shows the RNA gel image produced by the Bioanalyzer. The electropherograms for both mRNA samples (Figure 1B,C) display a main peak at approximately 1900 nucleotides, consistent with the expected length of the intact transcript (1922 nt). A secondary, considerably less intense peak is observed at ≈3800 nt and is most plausibly attributable to dimeric mRNA species. Notably, no peaks corresponding to shorter oligonucleotides are present, indicating the absence of degradation products in both preparations.

The calculated mRNA concentrations were 1210 ± 110 µg/mL for the unmodified transcript (88% monomer, 12% dimer) and 1747 ± 67 µ g/mL for 5moU-mRNA (85% monomer, 15% dimer), with relative errors of 9.1% and 3.8%, respectively.

### 2.3. Digital RT-PCR

Digital polymerase chain reaction (dPCR) is based on the amplification of the target DNA molecule (as in quantitative PCR) where the sample is partitioned into thousands of individual, physically separated, reaction compartments. These can be either liquid droplets in droplet dPCR or nano wells within a chip in chamber dPCR. This allows for the absolute quantification of nucleic acids by reducing the reaction into a “digital” format (positive or negative) without the need for standard curves as in traditional quantitative PCR [20,21].

In the case of RNA measurement, a reverse transcription (RT) step is required to convert RNA into complementary DNA (cDNA) prior to PCR amplification. This reverse transcription step can introduce variabilities in quantification, depending on factors such as the reverse transcriptase enzymes and the priming strategies employed (e.g., oligo dT primers, random hexamers, or gene-specific primers) [22].

Two approaches exist for integrating RT with dPCR. In one-step dPCR, the RT reaction and PCR amplification are combined in a single tube, minimising handling and reducing the risk of contamination. In two-step dPCR, the RT reaction is performed first, and the resulting cDNA is then transferred to a separate tube for PCR amplification, offering greater flexibility in downstream processing.

To quantify the mRNA samples by dPCR, we developed two distinct assays (JRC-LUC01 and JRC-LUC02) targeting different regions of the mRNA-FLUC sequence (Table 2).

The JRC-LUC01 was designed to amplify a sequence within the luciferase synthetic ORF. This method amplifies a 100 bp-long sequence stretch with 18 uracil residues and a GC content of 51%. Conversely, the JRC-LUC02 method was developed to amplify the 3′-end untranslated region (UTR) present in the synthetic RNAs. The amplicon generated by the JRC-LUC02 is 101 bp long, with 28 U residues (a 55.6% increase compared to JRC-LUC01) and a GC content of 55%.

While the two amplicons have similar lengths and predicted melting temperature (83.5 °C and 81 °C, respectively), they have significantly different levels of stability in their predicted secondary structures. The amplicon generated by the JRC-LUC01 assay has a ΔG of −18.8 (KJ/mol), whereas the JRC-LUC02 amplicon is more stable, with a ΔG of −34.3 (KJ/mol). The higher stability of the JRC-LUC02 amplicon is expected, as it covers the part of the 3′-UTR that is known to include secondary structures involved in a range of cellular and molecular functions [23].

To quantify the concentration of the RNAs (expressed as copy number/µL), the two RNA molecules were analysed by chamber dPCR on five different days using the JRC-LUC01 and JRC-LUC02 assays. Each sample was analysed in five replicates.

**Figure 2:**
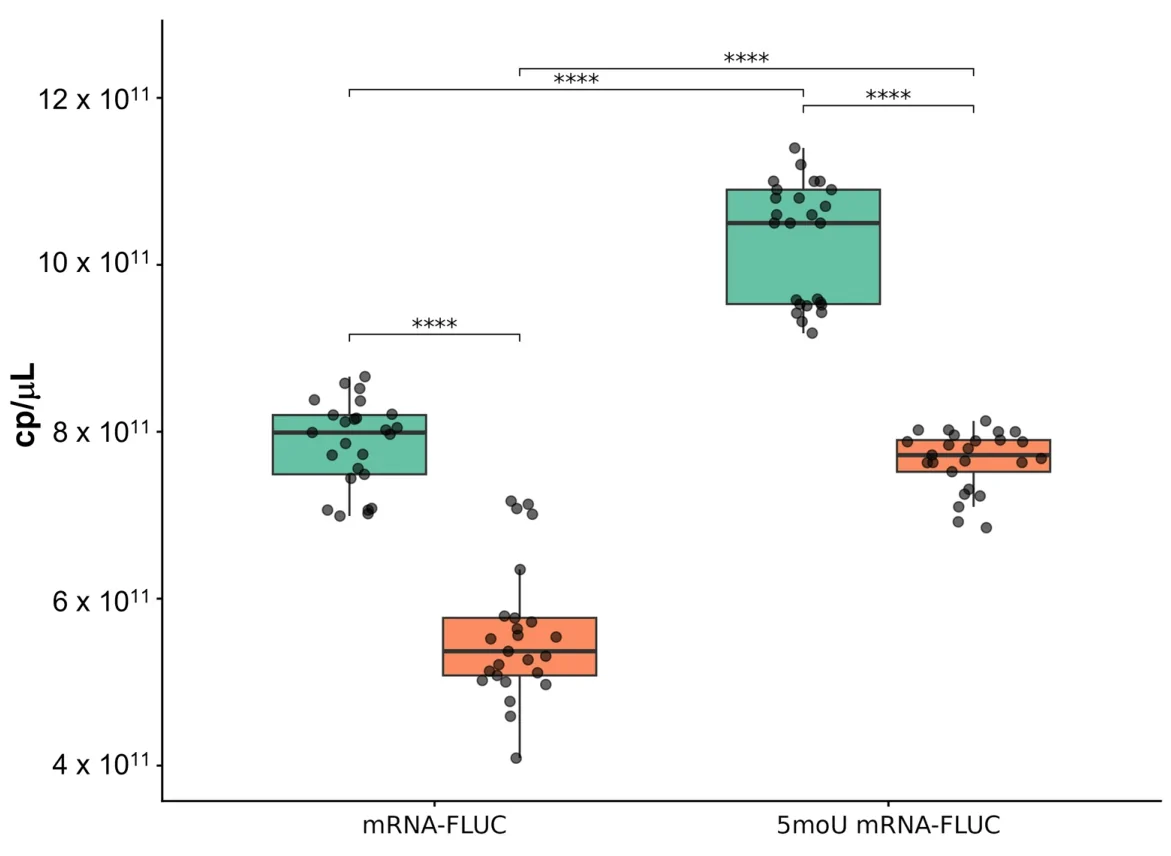
Quantitative results (RNA copies/µL) of JRC-LUC01 (green) and JRC-LUC02 (orange) of CleanCap mRNA-FLUC and CleanCap 5moU mRNA-FLUC by one-step dPCR. Samples were diluted 1:10,000 in carrier RNA to ensure stability and fall within the RT-dPCR quantification range, then stored at −70 °C till use. On the day of the measurement, one aliquot was further diluted 1:10,000 and analysed on the QIAcuity chamber dPCR system using both the JRC-LUC01 and JRC-LUC02 assays. The experiment was repeated over five consecutive days, with each sample measured in five replicates per run (n =25). A *t*-Test confirmed that the observed differences are statistically significant **** *p* < 0.0001.

Results are presented as mean ± standard deviation (SD) in Table 3 (n = 25) and as individual data points with mean ± SD in Figure 2. Digital PCR achieved reproducible mRNA quantification, with variability ranging from 5% to 15% (Table 3). Nevertheless, assays targeting different regions of the same mRNA molecule yielded statistically significant differences in quantification, with the JRC-LUC01 assay consistently reporting higher concentrations than JRC-LUC02 for both unmodified and 5moU-modified mRNA preparations (Figure 2).

Digital PCR provides results expressed as copy numbers per unit volume (e.g., cp/μL). To convert these values into a mass/volume metric, it is essential to know the molecular weight of the mRNA molecule. Using the predicted MW of 621 KDa for mRNA-FLUC and 629 KDa for 5moU mRNA-FLUC, together with the copy number values obtained from the JRC-LUC01 assay and one-step chamber dPCR, we calculated concentrations of 809 ± 56 µg/mL for the unmodified mRNA and 1076 ± 79 µg/mL for the 5moU-mRNA.

We next investigated how different digital PCR platforms may influence the mRNA quantification. Both mRNAs were measured on droplet dPCR and chamber dPCR systems.

**Figure 3:**
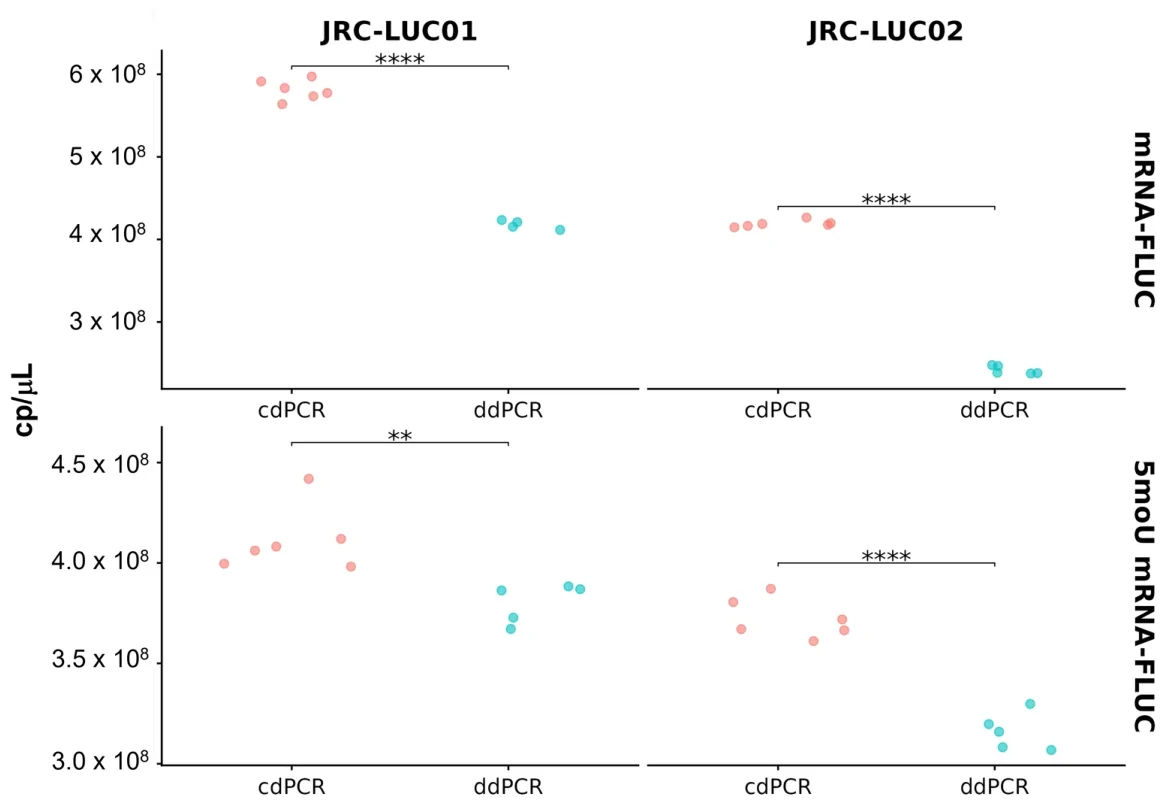
Comparison of assay performance (copies/µL) between one-step chamber (orange) and droplet (light blue) dPCR for JRC-LUC01 (left) and JRC-LUC02 (right) applied to unmodified mRNA and 5moU-modified mRNA. Each sample was measured in three replicates. A *t*-test confirmed that the differences are statistically significant (**** *p* < 0.0001; ** *p* < 0.01).

While measurements with a given instrument were highly reproducible and precise (CV < 2%), the absolute copies number obtained differed markedly between the two platforms (Figure 3). Specifically, the chamber-based instrument reported copy numbers 10% and 40% higher than those from the droplet-based instrument. This pattern was observed for both unmodified and 5moU-modified mRNAs and for both the JRC-LUC01 and JRC-LUC02 assays.

Multiple factors may account for the substantial variance observed between droplet-based and chamber-based one-step dPCR platforms, including the use of proprietary reverse transcriptase enzymes and different protocols across instruments. Several studies have evaluated the comparability of dPCR results across instruments and laboratories. In 2015, Dong and colleagues demonstrated, using a certified reference material, that measurements from different instruments became comparable once biases in partition volume were corrected [24]. Later, in 2017, Pavšič and co-workers conducted an interlaboratory study using both whole virus samples and extracted DNA, reporting that the variability between different instruments within the same laboratory was comparable to that observed when the same instrument was used across different laboratories [25].

**Figure 4:**
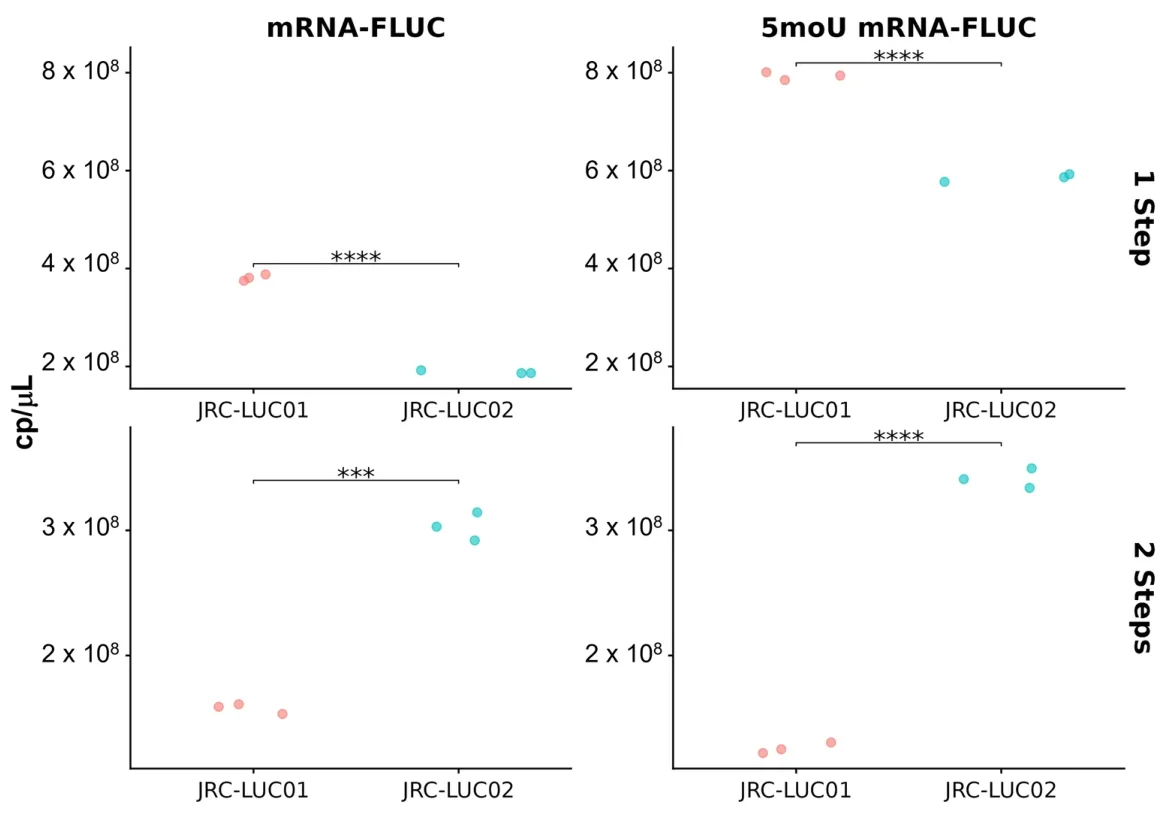
Comparison of assay performance (copies/µL) between one-step (1 Step) and two-step (2 Steps) ddPCR for JRC-LUC01 (orange) and JRC-LUC02 (light blue) applied to mRNA-FLUC and 5moU-modified mRNA-FLUC. Each sample was measured in three replicates. A *t*-test confirmed that the differences are statistically significant (*** *p* < 0.001; **** *p* < 0.0001).

We next evaluated whether one-step versus two-step PCR affects mRNA quantification using digital droplet PCR. The one-step and two-step dPCR approaches yielded statistically significant differences for both unmodified and 5moU-mRNAs (Figure 4). Furthermore, the one-step ddPCR assay yielded higher quantification values for JRC-LUC01 compared with JRC-LUC02 for both unmodified mRNA and 5moU-mRNA. In contrast, the two-step ddPCR assay showed the opposite trend, with JRC-LUC02 yielding higher values than JRC-LUC01.

In one-step d-PCR, the sample is partitioned before reverse transcription. As a result, even if multiple cDNA copies are generated from a single RNA molecule, they remain confined within the same partition and produce only one positive signal. This means one-step dPCR is unlikely to overestimate RNA copy numbers. On the contrary, because reverse transcription is not perfectly efficient, it may underestimate the true RNA concentration [26]. In contrast, two-step RT-dPCR performs reverse transcription separately before partitioning. This separation allows each step to be optimised independently, potentially improving reverse transcription efficiency. However, it also introduces the possibility that multiple cDNA molecules are generated from a single RNA template and distributed across different partitions, which can lead to overestimation of the actual RNA concentration. Studies comparing these two approaches have shown mixed inconsistent results [22,27,28,29]. Using Isotope Dilution Mass Spectrometry as an orthogonal primary measurement procedure, it was found that one-step dPCR gives approximately 10% lower concentration than two-step dPCR, suggestive of incomplete reverse transcription [22]. Of note, RT-efficiency may also be influenced by the choice of reverse transcriptase used for the reaction [30]. Comparative studies, such as those by Casmil et al., Falak et al., Malla et al., Niu et al., Sanders et al., and Pinheiro-de-Oliveira et al., have reported mixed results regarding which approach yields higher measured concentrations [20,22,27,28,29,31].

### 2.4. HPLC-UV

We used ion pair reverse-phase high-performance liquid chromatography (IP-RP-HPLC) equipped with UV detection at 260 nm to separate the different mRNA samples. Calibration curves were generated for each sample, allowing us to assess the quantitative performance of IP-RP-HPLC for measuring the amount of intact mRNA.

The elution programme developed enabled clear resolution of the intact-mRNA peak; in combination with the calibration curves this allows accurate quantification of the full-length transcript while effectively discriminating it from possible dimers or degradation fragments.

**Figure 5:**
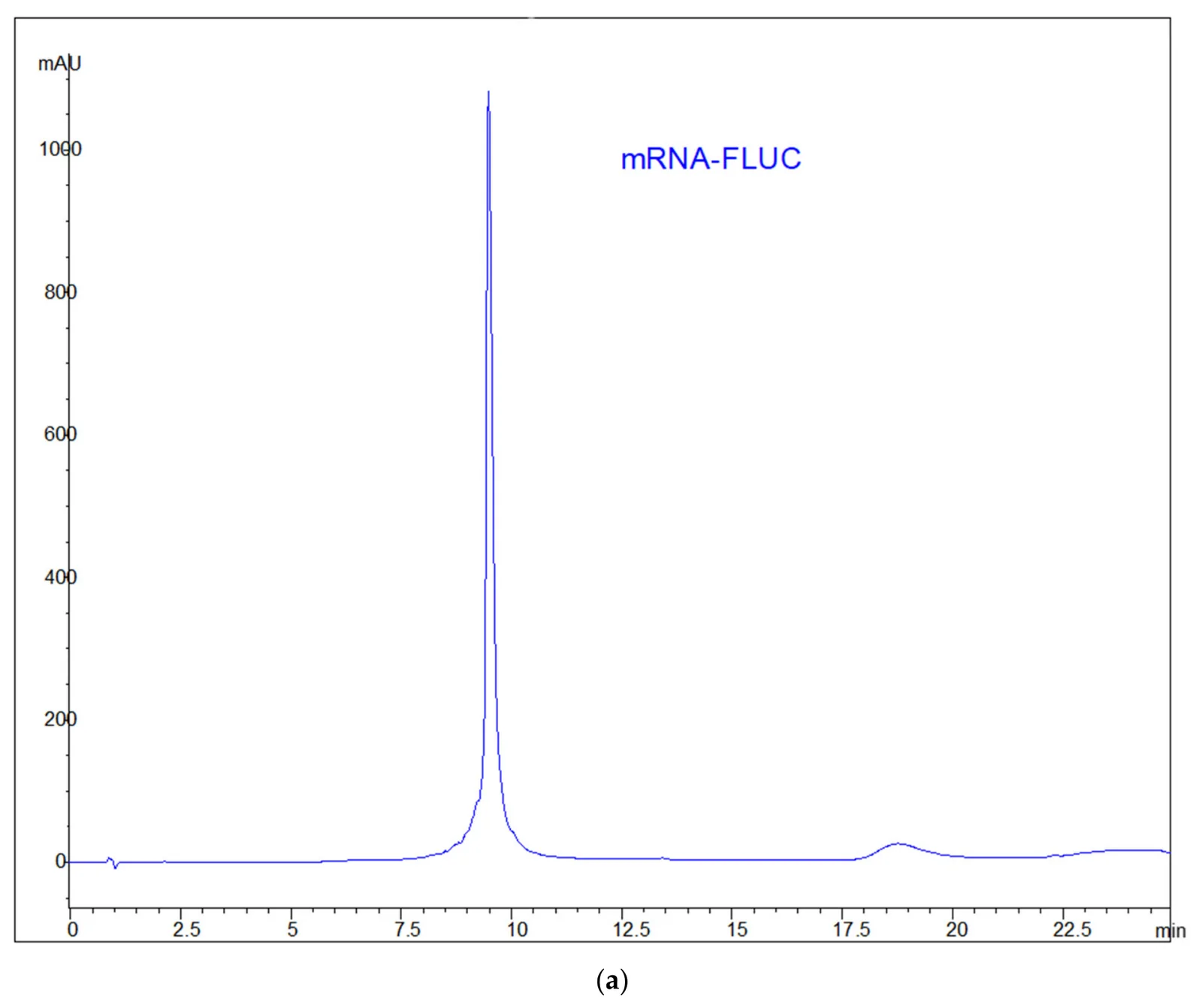

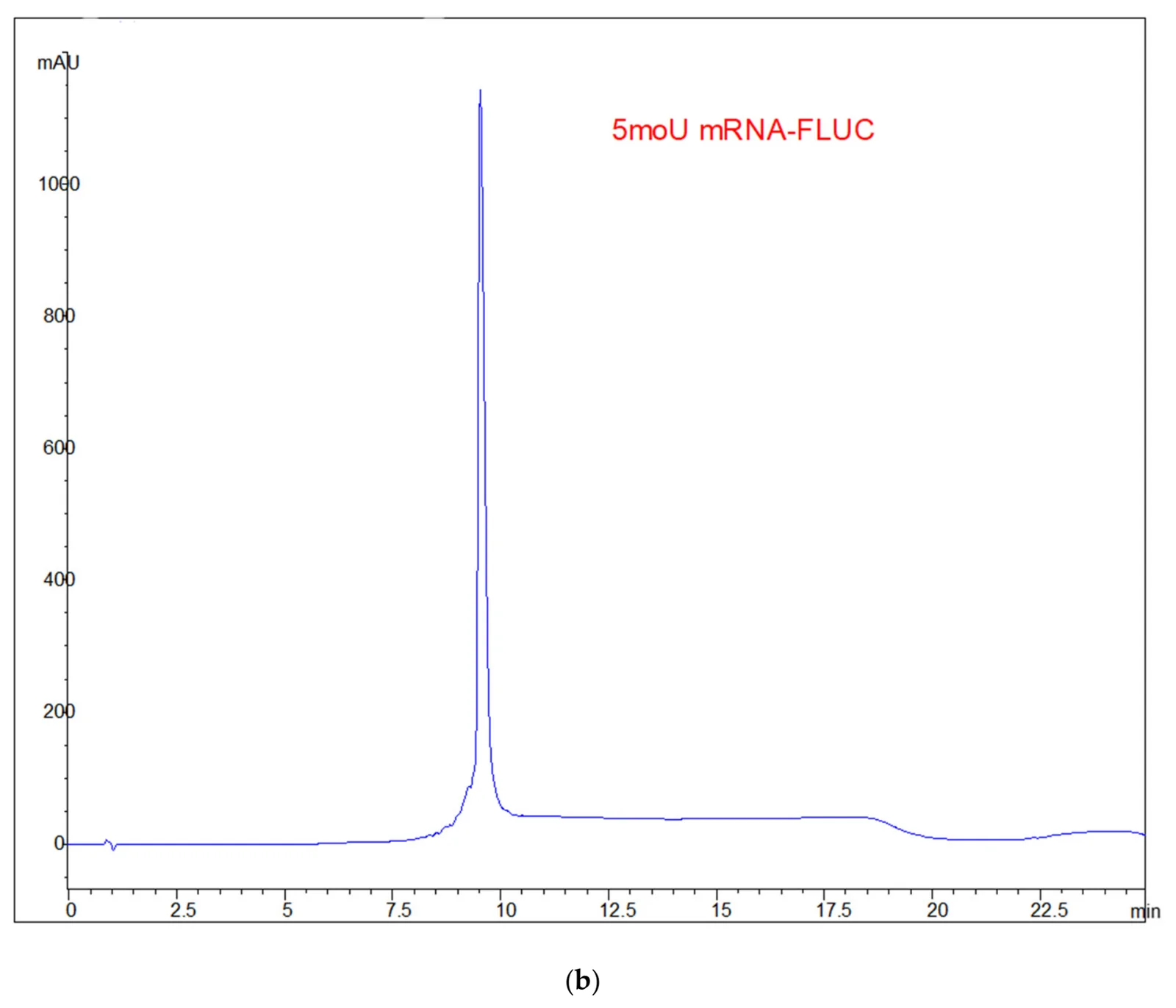
Representative images of IP-RP-HPLC chromatograms: (**a**) normal mRNA; (**b**) 5moU-mRNA.

The elution time from the IP-RP-HPLC column is directly related to the size of the RNA molecules. Figure 5a,b reports the IP-RP-HPLC chromatogram of the two mRNA samples. The single strongly retained peak corresponds to the full-length mRNA (≈1922 nt). No detectable signal is observed at the very early retention times where short degradation fragments would appear if present. The absence of early-eluting peaks therefore confirms that the preparations are free from appreciable fragmentation, in agreement with the electrophoresis results that indicated intact mRNA. Figure 5 also shows that the mRNA-FLUC and the 5moU mRNA-FLUC samples have identical retention times.

To quantify the amount of mRNA, a calibration curve, covering the range from 1 to 100 µg/mL, was generated using serial dilutions of the mRNA samples (1 mg/mL declared concentration), where the response (peak area) was plotted against the corresponding concentrations. The resulting calibration curve demonstrated a linear relationship (R^2^ > 0.99) across the tested range, confirming the method’s suitability for quantitative analysis.

The linear regression fit of the five-point calibration curve gave the equation y = 0.0098x + 9.5; This equation allows for the concentration of unknown samples to be calculated from the peak area. The concentration of 23.5 μg/mL for the incognito sample shows a difference of 6% compared to the expected value of 25 µg/mL. Considering that this sample has been diluted 40 times, it corresponds to a concentration of 944 µg/mL, in good agreement with the nominal 1 mg/mL value within experimental uncertainty.

## 3. Discussion

The rapid deployment of lipid-nanoparticle-encapsulated mRNA (LNP-mRNA) vaccines during the COVID-19 pandemic has highlighted the critical importance of robust analytical control strategies for these complex formulations. Regulatory agencies such as FDA, USP, EMA and European Pharmacopeia now require that key quality attributes of the mRNA drug substance (identity, purity, quantity and integrity) be measured.

Because no single analytical technique can simultaneously address all these attributes, the field relies on an orthogonal suite of methods, each with its own strengths and limitations. In this context, we set out to compare four widely used, commercially available platforms (UV spectroscopy, capillary electrophoresis, digital PCR and ion-pair reversed-phase HPLC with UV detection) using well-characterised Firefly luciferase mRNA (both unmodified and 5-methoxyuridine-modified) as study model materials. The aim was to generate a systematic, side-by-side assessment of their performance for the quantification of mRNA (sensitivity, sample consumption, throughput, and the ability to provide additional information on identity or integrity).

UV-based spectrophotometry is the most straightforward and widely available technique for nucleic-acid quantification in routine analytical laboratories. In our hands, the method shows excellent precision (reproducibility typically better than 5% relative error) but its accuracy is difficult to assess and strongly dependent on the value of the absorbance-to-mass conversion factor. Depending on whether the conventional value of 1 OD_260_ = 40 µg/mL or the more recent estimate of 1 OD_260_ = 37 µg/mL is used, the calculated concentration can differ by roughly 10%, introducing a substantial source of systematic uncertainty. In addition, the extinction coefficient is sequence-dependent; the development of sequence-specific tools, such as the mRNACalc webserver [32], offers a promising alternative by calculating ε_260_ based on the precise nucleotide composition. However, practical limitations persist, particularly for proprietary mRNA constructs where sequence details are restricted by non-disclosure agreements. Additionally, these tools assume complete hydrolytic conversion of mRNA into nucleoside monophosphates, which may not always be feasible or accurate in real-world applications. These considerations highlight the need for a critical assessment of results of UV-based measurements: at a minimum the OD_260_ value used for the conversion should always be explicitly reported. This is particularly relevant for NanoDrop measurements, where the conversion factor is pre-programmed and generally cannot be changed by the user.

Results also show systematic higher quantities (10% to 20%) for the 5moU-mRNA compared to unmodified mRNA. This could be due both to a real increase in the amount of FLUC-5moU-mRNA and also to differences in the ε_260_ extinction coefficient. Nucleoside modifications might alter UV absorbance properties, for example, due to structural changes (e.g., altered base stacking or secondary structure) that influence the extinction coefficient.

For capillary electrophoresis measurements, we employed a microfluidics-based instrument that combines separation, staining, and detection on a single chip. Samples are loaded into wells, and upon applying high voltage, molecules are separated based on size and charge. The system is cost-effective and suited for the sizing, quantification, and quality control of nucleic acids. Detection relies on fluorophore binding, with peak areas converted to RNA mass using proprietary standards and internal calibration. The assay requires only 1 µL of sample (within the 50–5000 pg/µL range) and involves a straightforward protocol. Overall, the Bioanalyzer offers a rapid, user-friendly, and highly sensitive approach to mRNA integrity assessment. Notably, the concentrations obtained by this method exceeded those measured by UV-based approaches, particularly for the 5moU-modified construct.

Digital PCR is by far the most sensitive technique to quantify mRNA; however, it is the most labour-intensive and technically demanding option. The 25 measurements performed across five different days yielded relative error ranging from 5% to 15%, demonstrating both high precision and excellent day-to-day reproducibility for the dPCR workflow. On the other hand, the absolute copy-number values obtained for the same mRNA preparation depended markedly on the assay employed for the quantification and consequentially on the targeted genomic region.

The JRC-LUC01 assay consistently gave results ≈30% higher than those obtained with the JRC-LUC02 assay (*p* < 0.001). This discrepancy can be attributed to the differing amplicon locations: JRC-LUC01 amplifies a 5′-proximal segment of the luciferase open-reading frame, whereas JRC-LUC02 targets the 3′-UTR junction. The 3′-UTR is predicted to adopt more stable secondary structures (ΔG ≈ −34 kJ mol^−1^ versus ΔG ≈ −19 kJ mol^−1^ for the LUC01 amplicon), a feature that has been shown to hinder reverse-transcriptase processivity and reduce cDNA synthesis efficiency [33,34]. Consequently, assays that interrogate structurally complex regions may underestimate the true mRNA copy number, underscoring the importance of assay design and target selection in digital-PCR quantification.

Our results show statistically significant differences between the two digital-PCR platforms (chamber-based versus droplet-based) and between the one-step and two-step workflows. These discrepancies can be traced to several mechanistic factors. First, each instrument uses a proprietary reverse-transcriptase (RT) enzyme; variations in enzyme processivity, thermostability and inhibitor tolerance inevitably affect the efficiency with which the mRNA template is converted into cDNA. Second, the priming strategy employed during RT differs between the workflows. In the one-step format, the RT step is coupled to the PCR amplification and relies on gene-specific primers that anneal only to the chosen amplicon. In contrast, the two-step protocol uses a mixture of oligo-dT and random hexamer primers, which initiates cDNA synthesis from the 3′ poly(A) tail and from multiple internal sites along the transcript. Because reverse transcription proceeds 5′ → 3′ from the primer-binding site, random-hexamer priming can enrich for the 3′ region of the mRNA, whereas the 5′ region may be under-represented if secondary structures impede polymerase progression. This bias explains the observation that, in the two-step assays, the JRC-LUC02 assay (which targets the 3′-UTR) yields higher copy numbers than JRC-LUC01 (which targets a 5′-proximal coding-region fragment). Similar effects of secondary structure on RT efficiency have been reported for complex 3′-UTRs [33,34]. Finally, differences in partitioning chemistry (droplet versus nanowell) and in the thermal-cycling parameters required by each platform can further modulate reaction kinetics, contributing to the observed platform-dependent copy-number variations. Overall, these findings underscore the importance of carefully standardising RT enzymes, priming strategies, and assay design when comparing absolute mRNA quantification across different dPCR technologies.

Results of the HPLC separation show that mRNA-FLUC and 5moU mRNA-FLUC sample have the same retention time. Overall, the method provides robust separation of intact mRNA, confirms the absence of fragmentation, and delivers accurate, linear quantification across the relevant concentration range. The technique has a reproducibility of ~5–10% and it requires only 2 μg of sample. While not the most sensitive, or straightforward method, it can be a practical alternative to capillary electrophoresis—particularly if already available in the laboratory—by providing both mRNA quantification and RNA integrity assessment. Additionally, the same column and method can potentially detect RNA-ionizable lipid adducts, in the final LNP-mRNA formulation enhancing its utility [35].

To summarise, Table 4 reports the mass concentration measured with the different techniques for the two mRNA samples. The data show that while the variance for each technique can be quite low (from a few percent for UV-based, dPCR and HPLC, to a maximum of 10% for the CE-based Bioanalyzer), the values can differ significantly between techniques.

A qualitative comparison of the different techniques for mRNA quantification is reported in Table 5.

## 4. Materials and Methods

### 4.1. Materials

CleanCap^®^ Firefly Luciferase (FLuc) mRNA, either modified with 5-methyluridine (5moU) (Cat. Num. L-7202-1000) or unmodified (Cat. Num. L-7602-1000) was obtained from Tebu-Bio (Roskilde, Denmark).

Both molecules were supplied as nominal 1.0 mg/mL solutions, each containing a reported sequence length of 1922 nucleotides with an open reading frame of 1661 nucleotides [36]. Using the sequence provided and the RNA Molecular Weight Calculator [37], the unmodified mRNA molecule has a predicted molecular weight of 621 KDa and the 5moU mRNA-FLUC a MW of 629 KDa.

The complete RNA sequence was kindly provided by Tebu-Bio via a Non-Disclosure Agreement.

According to the manufacturer’s specifications, the sample concentration is based on the absorbance measurements at 260 nm using the standard conversion factor of 40 μg/OD260.

Given the lack of certified reference standards for mRNA, the values reported represent comparative method performance rather than true analytical accuracy against an absolute reference.

### 4.2. UV Spectrophotometry

For UV-VIS, measurements were performed on a Thermo Fisher Evolution 350 UV-Vis spectrophotometer (Thermo Fisher Scientific Inc., Waltham, MA, USA) using micro quartz cuvettes of 1 cm optical pathlength. Samples were diluted in nuclease-free water, and absorbance measurements were recorded across a wavelength range of 200–800 nm at a scan speed of 400 nm/min.

Absorbance values at 260 nm were subtracted by the baseline readout at 350 nm. Average values and standard deviations were calculated using the Origin 2025 software, averaging three independent measurements. mRNA samples of nominal 1 mg/mL concentration were diluted between 40 and 1600 times for the different measurements.

### 4.3. NanoDrop

For concentration and purity assessment of the mRNA samples, a Thermo Fisher NanoDrop 2000/One spectrophotometer (Thermo Fisher Scientific, Waltham, MA, USA) was employed. A 1 μL aliquot of each undiluted mRNA sample was loaded onto the measurement pedestal, and absorbance was recorded at 260 nm (A260) to determine nucleic acid concentration. Three independent measurements were averaged using Origin software to find the mean values and standard deviations.

### 4.4. Bioanalyzer

The Agilent Bioanalyzer 2100 (Agilent, Santa Clara, CA, USA), a microfluidics-based capillary electrophoresis (CE) system, was used to assess both mRNA quantity and integrity. The RNA 6000 Nano Kit (Agilent, Santa Clara, CA, USA) was employed for analysis. Samples were prepared according to the manufacturer’s instructions, and 1 µL of each mRNA sample was loaded onto the instrument. The electropherograms were analysed using the Agilent 2100 Expert Software (v. B.02.10.SI764). Average values and standard deviations have been calculated using the Origin 2025 software, averaging three independent measurements.

### 4.5. Digital PCR

The droplet digital PCR (ddPCR) experiments were performed on a Biorad QX600 system (Biorad, Hercules, CA, USA).

One-step RT-dPCR reactions were carried out with Biorad One-Step RT-dPCR Advanced kit for probes (Biorad, Hercules, CA, USA). The final reaction volume of 22 µL contained 5.5 µL Supermix (Biorad), 2.2 µL reverse transcriptase (20 U/µL, Biorad), 1.1 µL of 300 nM DTT, primers and probes (400 nM/150 nM for FAM probe and 400 nM/50 nM for VIC probe), nuclease-free water and 5.5 µL of sample. Samples were diluted (10^4^) according to the dynamic range for dPCR in carrier RNA solution (10 ng/µL) and were analysed as at least three technical replicates within each run, including a non-template control. Droplets were generated with the Automated droplet generator and the reactions were carried out in a thermal cycler (Biorad) under the following conditions: 60 min reverse transcription at 50 °C and 10 min enzyme activation at 95 °C followed by 45 cycles using a two-step thermal profile of 30 s denaturation at 95 °C and 60 s annealing and extension at 60 °C, followed by 10 min of enzyme deactivation at 98 °C and then cooling at 4 °C. Following the amplifications, PCR plates were analysed on a droplet reader (QX600 Biorad), and data collected and analysed using the QX Manager software (Biorad, v. 2.2.0.71).

The two-step dPCR was carried out using the QuantiTect Reverse Transcription Kit (Qiagen, Venlo, The Netherlands) in accordance with the manufacturers’ instruction. Briefly, RNA was reverse transcribed by incubation for 15 min at 42 °C with an optimised mix of oligo-dT and random primers, which ensures cDNA synthesis from all regions of the transcript and yields a high number of templates. The resulting cDNA was diluted prior to dPCR. Reactions employed the Supermix for probes (Biorad) kit, and the thermal cycling programme was identical to that used for the one-step assay, apart from the additional reverse transcription step.

The chamber-based dPCR (cdPCR) experiments were performed on a QIAcuity Four dPCR system (QIAGEN), using a 26K 24-well nanoplate. Reactions employed the QIAcuity One-Step Advanced PCR kit (QIAGEN) in a final volume of 40 µL, comprising 10 µL of 4× One-Step advanced Probe Master Mix, 0.4 µL of 100× One-step Advanced RT Mix, 400 nM of each primer 150 nM of the FAM labelled probe -or 50 nM of VIC probe-, and 5 µL of template and nuclease-free water to volume. Thermal cycling conditions consisted of reverse transcriptase at 50 °C for 40 min and an initial denaturation at 95 °C for 2 min, followed by 45 cycles of 95° C for 5 s and 60 °C for 30 s. Samples were considered positive when more than two positive partitions displayed fluorescence, as described by [38,39]. Data was collected and analysed with the QIAcuity Software Suite 3.1.0.0.

Statistical analyses and visualisations of the PCR experiments were performed with the R software (R Core Team 2021) (v. 4.5.0) using standard libraries and the ggplot2 package (v. 3.5.2).

### 4.6. Ion Pair Reverse-Phase High-Performance Liquid Chromatography (IP-RP-HPLC)

Sample separation was performed on an HPLC system (Agilent Technologies, Series 1100, Santa Clara, CA, USA) equipped with a binary pump (G1312A), diode array detector (DAD; G1315B), and column oven (G1316A). Separation was achieved using a DNAPac RP column (2.1 × 100 mm, 4 µm particle size; product code 088923, Thermo Fisher Scientific) at a column temperature of 65 °C and a flow rate of 0.35 mL/min.

The mobile phase consisted of two components: mobile phase A containing dibutylammonium acetate (50 mM; TCI America, Portland, OR, USA) and triethylammonium acetate (100 mM; Sigma-Aldrich, St. Louis, MO, USA), and mobile phase B containing 50% acetonitrile (Sigma-Aldrich), dibutylammonium acetate (50 mM), and triethylammonium acetate (100 mM). The following gradient program was used: 0–1.5 min, 25% B; 1.5–4.5 min, 25–55% B; 4.5–19 min, 55–61% B; 19–19.5 min, 61–100% B; 19.5–25 min, 100% B. This was followed by a 5 min re-equilibration at 25% B.

The injection volume was 20 µL, corresponding to approximately 2 µg of mRNA per injection. Samples were prepared by diluting mRNA in RNase-free water. A five-point calibration curve was constructed, covering the range of 1–100 µg/mL, yielding the linear regression equation y = 0.0098x + 9.5 (R^2^ > 0.99). A blinded verification sample was prepared at a nominal concentration of 25 µg/mL as an independent quality control standard, not part of the calibration set. The ‘expected value’ of 25 µg/mL corresponds to the nominal preparation concentration of this verification sample, which was unknown to the operator during HPLC analysis.

Data acquisition and processing were performed using Agilent ChemStation software (version A.10.02).

## 5. Conclusions

Accurate quantification of mRNA drug substance is a key requirement for the development or manufacturing of mRNA therapeutics. Due to the lack of an mRNA reference material for quantification, it is difficult to assess how much each measurement technique deviates from the “true value”. UV-VIS (both cuvette-base and NanoDrop) results strongly depend on the extinction coefficient used for the conversion from absorbance measurement to mass concentration; the most sensitive technique (dPCR) strongly depends on the region used for amplification, the type of instrument, and the performance of the reverse transcriptase enzyme. In addition, differences in the quantification of modified mRNA may be expected.

All the techniques we tested for the quantification of mRNA are quite precise and reproducible (except for the Bioanalyser), but there are issues with their absolute accuracy: this is not surprising given the complexity of the mRNA molecule and the lack of certified reference materials. In fact, it is well established that both amplification efficiency and extinction coefficients depend not only on the sequence of the bases, but also on secondary and tertiary structures, which are, at present, difficult to measure or predict accurately. Nevertheless, our results demonstrate that all the methods evaluated, except for the Bioanalyzer, are highly reproducible and are in good agreement.

For the quantification of mRNA, UV-based measurements are the simplest and less technically demanding, offering good precision. Other techniques are more complex but provide additional quality attributes: the Bioanalyzer and HPLC can assess mRNA integrity, while dPCR can confirm identity through sequence-specific detection (see Table 5).

Overall, these considerations highlight the importance of using a consistent technique with a fixed measurement protocol when performing the relative quantification of mRNA across different synthetic batches. For absolute quantification, an uncertainty of approximately 10–15% (and likely higher) should be taken into consideration. These combined sources of uncertainty underscore the need for orthogonal measurements and, ultimately, for a universally accepted mRNA reference standard.

## Figures and Tables

**Table 1:** Calculated RNA mass concentration (µg/mL). ^(^*^)^ Ɛ = 40 µg/mL calculated using the same concentration factor as NanoDrop (i.e., 40 µg/mL). ^(^**^)^ Ɛ = 37 µg/mL. Each sample had a nominal concentration of 1 mg/mL and was measured in three replicates.

	mRNA-FLUC	5moU mRNA-FLUC
UV-Vis *	1012 ± 5	1268 ± 4
UV-Vis **	936 ± 5	1170 ± 4
NanoDrop	959 ± 3	1057 ± 12

**Table 2:** Details of the design of the two amplification assays for mRNA dPCR quantification.

Assay	Primer/Probe	Sequence	Amplicon Position
JRC-LUC01	LUC01-fw	ATCCTGAACGTGCAGAAGAAGC	406–505
	LUC01-rev	TCACGAAGGTGTACATGCTCTG	
	LUC01-Probe-FAM	ACAGCAAGACCGACTACCAGGGC	
JRC-LUC02	LUC02-fw	ATCCTGATCAAGGCCAAGAAGG	1612–1710
	LUC02-rev	CAAGGGAGAGAAGAAGGGCATG	
	LUC02-Probe-VIC	TAAGCTGCCTTCTGCGGGGCTT	

**Table 3:** Performance of the two assays by one-step, chamber-based dPCR. RSD: Relative Standard Deviation.

Sample	JRC-Assay	Results cp/µL (Mean ± SD)	RSD
mRNA-FLUC	LUC01	(7.85 ± 0.55) × 10^11^	7%
5moU mRNA-FLUC	LUC01	(10.30 ± 0.77) × 10^11^	8%
mRNA-FLUC	LUC02	(5.57 ± 0.81) × 10^11^	15%
5moU mRNA-FLUC	LUC02	(7.66 ± 0.35) × 10^11^	5%

**Table 4:** Calculated RNA mass concentration (µg/mL) from the different techniques. ^(^*^)^ Ɛ = 40 µg/mL calculated using the same concentration factor as NanoDrop (i.e., 40 µg/mL); ^(^**^)^ Ɛ = 37 µg/mL.

	mRNA-FLUC	5moU mRNA-FLUC
UV-Vis *	1012 ± 5	1268 ± 4
UV-Vis **	936 ± 5	1170 ± 4
NanoDrop	959 ± 3	1057 ± 12
Bioanalyzer	1210 ± 110	1747 ± 67
dPCR	809 ± 56	1076 ± 79
HPLC-UV	944 ± 28	1195 ± 36

**Table 5:** Qualitative evaluation of mRNA quantification techniques.

Technique	Typical Measurand	Reported Precision	Main Strengths	Main Limitations	Potential Use Cases
UV spectrophotometry (cuvette-based)	mg/mL	<5%	Low cost, GLP-compatible, simple operation	Requires relatively large sample volumes (~400 µL) and relies on an accurate extinction coefficient; less suitable for scarce samples	Concentration measurement in GLP/GMP environment
UV spectrophotometry (NanoDrop)	mg/mL	<5%	Small sample volume (1 µL) Low cost Simple and fast	Fixed conversion factor (potential bias)	Routine concentration check of abundant RNA
Bioanalyzer (capillary electrophoresis)	mg/mL	≈10%	Small sample volume (1 µL), provides RNA integrity information and fragment size distribution; fast and sensitive	Precision is modest compared with other methods; consumable costs are higher than simple spectrophotometry.	Assessment of RNA integrity before downstream applications
Digital PCR (dPCR)	Copies/µL	≈10%	Absolute quantification without standard curve, very high sensitivity, sequence-specific detection, can confirm mRNA identity	Instrument and kit dependent typically requires large dilutions (≈10^8^-fold); expensive and technically complex	Absolute quantification of a low-abundance transcript
HPLC-UV	mg/mL	≈10%	Linear calibration, can resolve mRNA fragments and RNA–lipid adducts.	Requires method optimisation, medium-level technical expertise, and a relatively large amount of material (≈2 µg)	Detection of degradation products or RNA–lipid conjugates

## Data Availability

The datasets that were generated for this study are available on request.
